# Health Tract, No. 177

**Published:** 1863-11

**Authors:** 


HEALTH TRACT, No. 177.

### CANCER

Is the Latin word for "Crab," and was applied to that kind of sore which has the spraggling look of that ugly animal. The essence of cancer is in a depraved condition of the blood; it is hard, soft, or yielding as a sponge; it is a loathsome and thus far an incurable disease. It is worse than incurable, because if healed up, or cut out at one place, it is sure to sprout up in a dozen others. Sometimes a sore is cured, that looks like a cancer, and the pretended curer is willing enough that it should be considered a real one, hence ingenious impositions have been practiced on many and many hearts sickened to death by false hopes. Cancer is developed in two ways almost always. First, nature makes an effort to pass out of the system, through some gland, matters, the presence of which is hurtful; if thwarted, the gland under certain conditions becomes cancerous, becomes an eating, running sore, which, if let alone, will always secure a longer life than if it is not allowed to run, by "healing it up," or cutting it out. Second, when a gland is injured by a cold settling in it, or by a bruise, cancerous disease begins to develop itself when the blood is in a depraved condition. The same cold or bruise would have passed off without injury, had the individual possessed vigorous health. Cancer is confined chiefly to females, because of their in-door life, so promotive of a poisoned blood from want of exercise and from the routine nature of their existence. Its commonest seat is the left breast, first appearing an undiscolored hard lump the size of a marble or pea, growing very slowly, and as it becomes more active, giving the characteristic star-like pains; pains which shoot out or lancinate in every direction like the rays of a star. Any pain of this sort, confined to one spot, should be always regarded with apprehension. After a while the skin assumes a puckered appearance, sometimes with heat, soon breaks and throws out a thin fluid, with more or less blood, next emitting a most offensive smell as the fungus mass springs forth and eats its horrible way into the very vitals.

Cancer of a more superficial character sometimes attacks the nose, the lower lip, and the corner of the eye, looking at first like a "fever-blister," or a wart with an uneven surface; at other times it comes with a dry scale, which falls, or is picked off; another and another comes, each going deeper, until the hateful sore assumes its characteristic appearance. It is admitted the world over, because statistical tables prove it, that "cutting out a cancer," especially from the breast, is fatal in nine cases out of ten. Whether that tenth case may not be a cancer only in appearance, is a question. As all acknowledge that cancer arises from a depraved condition of the blood, those who fear cancer, with or without cause, should use means to keep the general system in the highest health possible, as a means of purifying the blood, and thus indefinitely postpone the breaking out of the cancerous sore; keeping it in its hard state as it were; just as tubercles in the lungs, which are hard lumps there, and which are not capable of causing common consumption as long as they remain hard, may be kept in abeyance for a long lifetime by a vigorous following out of those activities which the experienced physician has so often seen to be efficient in such cases. Meanwhile, if any person has an actual sore which seems to be of a cancerous character, try any body and any thing reasonably promising even a slight benefit.

